# Stimulating research on childhood adversities, borderline personality disorder, and complex post-traumatic stress disorder

**DOI:** 10.1186/s40479-021-00152-y

**Published:** 2021-03-30

**Authors:** Annegret Krause-Utz

**Affiliations:** 1 Institute of Psychology, Department of Clinical Psychology, Leiden University, Leiden, The Netherlands; 2 Leiden Institute for Brain and Cognition, Leiden University, Leiden, The Netherlands

**Keywords:** Borderline personality disorder, Child sexual abuse, Complex trauma, Complex post-traumatic stress disorder, Post-traumatic stress disorder

## Abstract

Traumatic events of a long-lasting invasive, primarily interpersonal nature (e.g., childhood abuse, intimate partner violence) can have wide-ranging consequences across various life domains. This involves an increased risk of developing mental disorders, such as (complex) post-traumatic stress (PTSD, CPTSD) and borderline personality disorder (BPD). Both within and outside of these diagnostic boundaries, complex trauma has been associated with emotional dysregulation, dissociation, distrust, interpersonal problems, and maladaptive coping behaviours, such as self-harm and suicidal behaviour. Knowledge on the devastating consequences of complex trauma has steadily increased. One of the remaining research questions is why some people develop certain psychopathological symptoms or disorders after complex trauma while others do not. Moreover, more research is needed to better understand how disorders such as BPD and CPTSD can be differentiated, both descriptively and empirically. This special issue brings together a collection of review articles and original research articles on this topic to stimulate further research in the field. Findings enhance the understanding of long-term consequences of childhood adversities and highlight important psychopathological mechanisms that may underlie an increased risk to develop certain mental disorders.

## Introduction

In the current literature, the term “complex trauma” is used to describe events of a long-lasting, invasive, and primarily interpersonal nature (e.g., severe child abuse and neglect by primary caregivers, intimate partner violence, rape, sex trafficking or sexual exploitation, medical trauma, war and refugee trauma, torture, and/or genocide), as opposed to single-incident trauma. It is still not entirely understood why some people develop certain psychopathological symptoms after traumatic events while others do not.

Experiencing traumatic events is not uncommon. In a large representative sample of 68 894 adults from 24 countries, over 70% of respondents reported a traumatic event [1, 2]. Approximately 3.9%-5.6% of trauma-exposed individuals develop post-traumatic stress disorder (PTSD) [3], which is characterized by traumatic re-experiencing, avoidance of traumatic reminders, and hypervigilance [4, 5].

In 2018, the 11^th^ International Classification of Diseases (ICD-11) introduced the diagnosis of complex post-traumatic stress disorder (CPTSD) as a “sibling diagnosis” of PTSD [4]. In addition to the three “classic” PTSD symptoms (traumatic re-experiencing, avoidance, hypervigilance), CPTSD is characterized by symptoms in three domains of disturbances in self-organization (DSO): 1) emotional dysregulation (e.g., problems in self-soothing and decreased stress tolerance), 2) interpersonal problems (e.g., avoidance of relationships), and 3) a negative self-concept (e.g., beliefs about oneself as being a failure) [4]. Since the publication of the CPTSD definition, research on this new diagnosis has steadily increased. While consensus regarding the validity of the CPTSD diagnosis is still lacking, there is growing evidence that CPTSD detects a distinct group of individuals with more severe impairment, who were exposed to multiple forms of trauma [6–8], mostly of an interpersonal nature [9, 10]. Empirical studies further supported the validity of the 6-factor symptom structure of CPTSD and its distinction from PTSD [6]. At the same time, researchers have raised the question whether CPTSD is indicative of a more severe form of psychological distress after trauma rather than a disparate constellation of symptoms (e.g., [11, 12]. The article by Andreas Maercker [13] gives a comprehensive overview of the development of the CPTSD diagnosis and highlights the growing attention to this research topic.

One of the remaining research questions is how CPTSD can be differentiated from borderline personality disorder (BPD), both descriptively and empirically. There is considerable symptom overlap, which has been addressed by a growing number of studies (e.g., [11, 14–17]. BPD is characterized by a marked instability in affect, cognitions, behaviour and interpersonal relationships [5]. Current conceptualizations propose that three core domains underlie the complexity and heterogeneity of BPD symptoms: emotion dysregulation including stress-related impulsivity (maladaptive coping behaviours, such as self-harm and suicidal attempts), disturbed self-image, and interpersonal disturbances [18]. Unlike CPTSD, the diagnosis of BPD does not require an index traumatic event. However, individuals with BPD often report a history of severe abuse and neglect, and rates of childhood trauma are higher than in other psychiatric conditions [19, 20].

In their review article, Julian D. Ford and Christine A. Courtois provide a comprehensive overview over the relationship and differences between CPTSD and BPD. Their article summarizes recent findings on prevalence and comorbidity, clinical phenomenology, association with traumatic antecedents, psychobiology, and psychotherapeutic treatment of the two disorders.

Thereby, the authors provide a conceptual framework which will stimulate further research on the topic.

Research articles, included in this special issue, shed more light on the consequences of childhood adversities. Seitz and colleagues [21] used eye-tracking to investigate hypersensitivity to negative, potentially threatening interpersonal cues in BPD and its association with adverse childhood experiences. Findings point towards a childhood trauma-related anger bias and a visual hypervigilance towards the eyes of facial expressions in patients with BPD. Future studies should shed light on the remaining question whether these findings are specific for BPD or related to a history of childhood abuse and neglect in a trans-diagnostic manner.

The study by Hepp and colleagues [22] tested whether individuals who experienced childhood abuse and neglect show higher levels of distrust and perceived threat. Participants played two rounds of a hypothetical distrust game and an emotion rating task with angry, fearful, and happy facial expressions. Results suggest higher levels of distrust and a weaker decrease in distrust following positive feedback in individuals with childhood trauma.

Two studies in this article collection focused on child sexual abuse (CSA). Collin-Vézina and colleagues [23] explored the associations between CSA and non-suicidal and suicidal self-injurious thoughts and behaviors in relation to CSA disclosure experience. This qualitative study highlights the reciprocal influences between CSA disclosure and maladaptive coping behaviors as an attempt to seek help, which has important clinical implications. The study by Krause-Utz and colleagues [24] revealed a significant association between self-reported CSA and sexual revictimization in adult intimate relationships. Borderline personality features, maladaptive cognitive coping styles (e.g., self-blame, rumination) and dissociation were found to be important factors in this relationship.

Deuter and colleagues [25] investigated testosterone reactivity to an acute psychosocial stressor (Trier Social Stress Test, TSST) in women with BPD, PTSD, both disorders, and healthy control. The authors found an increase in testosterone after acute stress exposure across all groups, independent of BPD or PTSD status, and discuss possible explanations for this finding.

All in all, this article collection highlights the growing attention to the wide-ranging consequences of complex trauma. More research is needed to understand the inter-relationships of PTSD, CPTSD, and BPD as well as differences between these diagnoses.

## Data Availability

Not applicable.
